# QuickStats

**Published:** 2015-06-19

**Authors:** FROM THE NATIONAL CENTER FOR HEALTH STATISTICS

**Figure:**
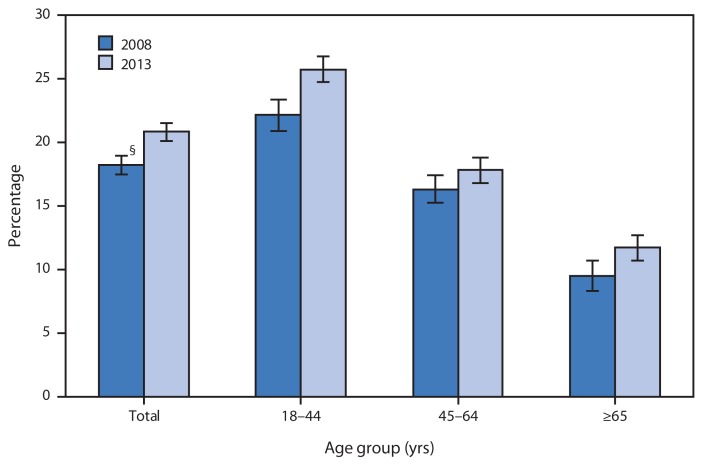
Percentage of Adults Aged =18 Years Who Met National Guidelines for Aerobic Activity and Muscle Strengthening,* by Age Group — National Health Interview Survey, United States, 2008 and 2013^†^ * Per U.S. Department of Health and Human Services *2008 Physical Activity Guidelines for Americans*. Available at http://www.health.gov/paguidelines/guidelines/default.aspx. Respondents defined as meeting both aerobic-activity and muscle-strengthening guidelines reported moderate-intensity physical activity for ≥150 minutes per week, vigorous-intensity physical activity for ≥75 minutes per week, or an equivalent combination of moderate- and vigorous-intensity activity, and engaging in physical activities specifically designed to strengthen muscles at least twice per week. ^†^ Estimates are based on household interviews of a sample of the civilian, noninstitutionalized U.S. population and are derived from the National Health Interview Survey sample adult component. ^§^ 95% confidence interval.

The percentage of adults aged ≥18 years who met the aerobic-activity and muscle-strengthening guidelines increased from 18.2% in 2008 to 20.8% in 2013. Adults aged 18–44 years were the most likely to meet the aerobic-activity and muscle-strengthening guidelines, and those aged ≥65 years were the least likely in both 2008 and 2013. For all age groups, the percentage meeting the guidelines increased from 2008 to 2013.

**Source:** CDC. National Health Interview Survey data, 2008 and 2013. Available at http://www.cdc.gov/nchs/nhis.htm.

**Reported by:** Travis Combest, MS; Sirin Yaemsiri, PhD, syaemsiri@cdc.gov, 301-458-4186; Deepthi Kandi.

